# Nitrogen Supply and Host-Plant Genotype Modulate the Transcriptomic Profile of *Plasmodiophora brassicae*

**DOI:** 10.3389/fmicb.2021.701067

**Published:** 2021-07-08

**Authors:** Kévin Gazengel, Yoann Aigu, Christine Lariagon, Mathilde Humeau, Antoine Gravot, Maria J. Manzanares-Dauleux, Stéphanie Daval

**Affiliations:** IGEPP, INRAE, Institut Agro, Université Rennes 1, Le Rheu, France

**Keywords:** clubroot, *Brassica napus*, host–pathogen interaction, abiotic stress, RNA-seq, root, pathogenicity-related genes

## Abstract

Nitrogen fertilization can affect the susceptibility of *Brassica napus* to the telluric pathogen *Plasmodiophora brassicae*. Our previous works highlighted that the influence of nitrogen can strongly vary regarding plant cultivar/pathogen strain combinations, but the underlying mechanisms are unknown. The present work aims to explore how nitrogen supply can affect the molecular physiology of *P. brassicae* through its life epidemiological cycle. A time-course transcriptome experiment was conducted to study the interaction, under two conditions of nitrogen supply, between isolate eH and two *B. napus* genotypes (Yudal and HD-018), harboring (or not harboring) low nitrogen-conditional resistance toward this isolate (respectively). *P. brassicae* transcriptional patterns were modulated by nitrogen supply, these modulations being dependent on both host-plant genotype and kinetic time. Functional analysis allowed the identification of *P. brassicae* genes expressed during the secondary phase of infection, which may play a role in the reduction of Yudal disease symptoms in low-nitrogen conditions. Candidate genes included pathogenicity-related genes (“NUDIX,” “carboxypeptidase,” and “NEP-proteins”) and genes associated to obligate biotrophic functions of *P. brassicae*. This work illustrates the importance of considering pathogen’s physiological responses to get a better understanding of the influence of abiotic factors on clubroot resistance/susceptibility.

## Introduction

Plants are exposed to a wide range of phytopathogens, such as fungi, bacteria, viruses, nematodes, or protists, which cause infectious diseases. The outcome of the plant/pathogen interaction can be influenced by multiple environmental factors, both biotic (e.g., plant-associated microbiota) and abiotic (e.g., temperature, light, water content, salinity, pH, or nitrogen). Nitrogen (N) is a central element in various metabolic pathways: synthesis of enzymes, coenzymes, amino acids, and proteins; photosynthesis; and respiration (Scheible et al., 2004; Xin et al., 2014). Then, the plant’s N status is a critical determinant not only for growth, development, and productivity, but also for plant response (susceptibility and resistance/tolerance) to both abiotic constraints [such as water (Zhong et al., 2017)] and plant pathogens (Mur et al., 2016). In general, it has been considered that the effect of N on plant diseases was dependent on the lifestyle of the pathogens (Solomon et al., 2003): the development of biotrophic pathogens would be enhanced by N, while the opposite effect would be observed for necrotrophic pathogens. In fact, the effect of N on plant disease, described in many examples, can be opposite depending on the pathogen and on the plant host. Thus, an increase in severity of infection by N fertilization was reported, for example, in powdery mildew (Zhang et al., 2019), stripe rust (Devadas et al., 2014), wheat blast (Ballini et al., 2013), and rice blast (Huang et al., 2017). On the contrary, disease decreases due to N nutrition were described in take-all (Brennan, 1992) and in leaf spot diseases (Krupinsky et al., 2007). Moreover, for the same pathogen, the situation may not be as clear-cut. For example, high N fertilization rates led to higher plant susceptibility to *Botrytis cinerea* in grape (R’houma et al., 1998), legumes (Davidson and Krysinska-Kaczmarek, 2007), sweet basil (Yermiyahu et al., 2006), and begonia (Pitchay et al., 2007), but to lesser symptoms in tomato in which infection also varied according to the pathogen isolates (Lecompte et al., 2010). In a review that summarized data from 114 studies, an increase, decrease, or no effect of N on plant disease was reported in 62, 42, and 10 cases, respectively (Sun et al., 2020). In addition, the different forms of N supply (ammonium NH_4_^+^ or nitrate NO_3_^–^) can have various effects on plant disease severity because of the use of different assimilation and metabolism pathways (Bolton and Thomma, 2008; Mur et al., 2016).

Plant N nutrition influences both constitutive and induced defenses, as an element in key signal molecules such as NO and polyamines (Takahashi, 2016, 2020). Different N conditions can modulate plant resistance based on pathogen-associated molecular patterns triggered immunity (PTI) and effector-triggered immunity (ETI) (Mur et al., 2016). N-biochemical-mediated defenses are achieved through plant metabolites (e.g., phytoalexins, antimicrobial proteins, amino acids, and organic acids) and defense-related enzymes (Ngadze et al., 2012; Wang et al., 2018). Plant N availability can also impact cellular structure and composition affecting the thickness of the plant’s physical barrier (Zhang et al., 2017). N input modulates plant defense but also impacts disease susceptibility by increasing the availability of N compounds for exploitation by pathogens (Tavernier et al., 2007), shaping the plant architecture and providing an environment favorable for pathogen growth and spore production (Mur et al., 2016). Although several studies reported the importance of N supply on pathogenesis and effector delivery (Soulie et al., 2020; Sun et al., 2020), conflicting results have been observed. Thus, in *Magnarpothe oryzae*, the induction or repression of some effectors was found under N starvation conditions (Donofrio et al., 2006; Huang et al., 2017).

The abundant literature highlights the various and contradictory effects of N fertilization on the outcome of the plant/pathogen interaction and underlines the need to better understand the mechanisms underlying this process in different pathosystems.

In the case of clubroot, a soilborne disease caused by the obligate parasite *Plasmodiophora brassicae* and affecting the Brassicaceae, the study of N effect is of prime importance, notably in oilseed rape (*Brassica napus*) since this crop requires the use of relatively high amounts of N fertilizer (Rathke et al., 2005). Resistance to plant pathogens and adaptation to low-input agricultural practices (especially low N input) are thus two major traits targeted in oilseed rape breeding efforts (Bouchet et al., 2014, 2016). In this pathosystem, several studies have suggested that a high N supply tends to reduce the damage caused by *P. brassicae* infection (Dixon, 2009; Gossen et al., 2014). On the other hand, a study on a set of 92 diverse oilseed rape accessions and 108 lines derived from a cross between “Darmor-*bzh*” (clubroot-resistant) and “Yudal” (clubroot-susceptible) showed that some genotypes classified as susceptible to clubroot became resistant under N constraint (Laperche et al., 2017). In this previous work, we have shown that N supply can modulate the effect of two QTLs controlling symptom development (Laperche et al., 2017) and spore production (Aigu et al., 2018). Surprisingly, no studies have been published about the effect of N directly on the clubroot pathogen *P. brassicae*, while knowing that N input could have an influence on many factors involved in the different steps of its epidemiological cycle. First, the resting spores, survival form in the soil, germinate when root exudates are sensed, leading to biflagellate primary zoospores that infect the root hairs. Then, zoospores multiply to form the primary plasmodia. During this primary phase of infection, metabolisms of cell wall chitin digestion, starch, citrate cycle, pentose phosphate pathway, pyruvate, trehalose, carbohydrates, and lipids are highly active (Schwelm et al., 2015a, b; Bi et al., 2016) making an interaction between carbon metabolism and N supply possible. In the secondary infection phase, secondary zoospores are released from the root hairs and directly invade host cortical cells, then forming the secondary plasmodia (Kageyama and Asano, 2009). During the second phase of infection, genes involved in basal and lipid metabolism (Bi et al., 2016), in signal transduction pathways (Bi et al., 2019), and in activation of cell differentiation, growth, reproduction, as well as genes coding for proto-oncogenes (Bi et al., 2018) are highly expressed. At this stage, *P. brassicae* forms galls in root because of hypertrophy and hyperplasia of infected roots by the modification of hormone levels (Siemens et al., 2006), leading to the obstruction of nutrient and water transport and then a reduction of plant growth. During both primary and secondary phases, it has been suggested that *P. brassicae* could secrete effectors, but the validation of their real roles in infection is still in progress (Kombrink and Thomma, 2013; Schwelm et al., 2015b; Rolfe et al., 2016; Zhang et al., 2016; Perez-Lopez et al., 2018; Daval et al., 2019, 2020), except for a predicted secreted methyltransferase interfering in the plant salicylic acid-induced defense (Ludwig-Muller et al., 2015).

Understanding how *P. brassicae* responds to N input in the different stages of its life cycle to influence infection is, then, of fundamental importance. In this context, the aim of the present study is to explore how N supply may influence the functions of the pathogen and its pathogenesis. For this, we followed clubroot disease progression through a time-course experiment [14, 27, and 42 days post-inoculation (dpi)] in two *B. napus* genotypes (Yudal and HD-018) under two N supply conditions (1 and 8 mM). The time-course allowed to analyze the N effect at both stages of the *P. brassicae* life cycle. The host genotypes were chosen because of their different responses to clubroot according to N nutrition: both were susceptible to clubroot in normal N supply (8 mM), and only the genotype Yudal showed a drastic reduction of the disease symptoms under N constraint (1 mM) (Laperche et al., 2017). To identify underlying molecular pathogen mechanisms potentially involved in the modulation of the disease by N input, we assessed (i) the phenotype of the two host-plant genotypes harboring different responses to N starvation and (ii) the transcriptome of the pathogen using RNA-sequencing (RNA-seq).

## Materials and Methods

### Plant Material and Pathogen Inoculation

Two oilseed rape genotypes, Yudal (called Y) and HD-018 (called HD), were used in this study: the *B. napus* cv. Yudal is a spring oilseed rape and HD-018 is a double haploid line from the cross between Darmor-*bzh* and Yudal (Laperche et al., 2017). The eH isolate of *P. brassicae* belonging to pathotype P1 (Some et al., 1996; Fahling et al., 2003; Daval et al., 2019) was used for inoculation.

Seeds from the two *B. napus* genotypes were sown in pots filled with “Falienor 922016F3” medium (65% Irish peat, 20% black peat, 15% perlite, and 2% clay). Pots were maintained in a phytotron at 22°C (day) and 19°C (night) with a 16-h photoperiod. Two Hoagland nutritive solutions were used, one containing 1 mM of nitrogen (named N1) and the other 8 mM (named N8) (Supplementary Table 1A), in which nitrogen was present only as nitrate (without ammonium), the preferred nitrogen source of oilseed rape. Pots were watered periodically at the bottom of the pots twice a week from sowing to 21 days after sowing, and then three times a week. This frequency was based on the requirement of the plants under N1 condition. Since transpiration and water requirement were higher in the N8-treated plants, those plants were also additionally supplied with water when needed, so as to avoid water stress. Plants were inoculated with a resting spore suspension of *P. brassicae* eH isolate. Non-inoculated (healthy) plants were also grown to make sure that no symptoms appeared and that the plants were growing well. For plant inoculation, clubs propagated on the universal susceptible host Chinese cabbage (*B. rapa* spp. *pekinensis* cv. Granaat) were collected, homogenized in a blender with sterile water, and separated by filtration through layers of cheesecloth. The resting spores were then separated by filtration through 500-, 100-, and 55-μm sieves to remove plant cell debris. The spore concentration was determined with a Malassez cell and adjusted to 1 × 10^7^ spores ml^–1^. Inoculation was done as described previously (Manzanares-Dauleux et al., 2000): 7-day-old seedlings were inoculated by pipetting 1 ml of the spore suspension onto the soil surface at the base of each seedling. Experiments were conducted in four replicates. Inoculated root samples for nucleic acid extraction were from 24 pooled plants at 14 dpi (primary phase of infection), 12 pooled plants at 27 dpi (secondary phase of infection), and 6 pooled plants at 42 dpi, for each combination of N level and genotype, and for each of the four replicates (total = 16 samples per time-course point, i.e., 48 in all). An additional six plants per modality were grown for aerial and root biomasses weighing.

### Phenotyping: Plant Characterization and Disease Assessment

At each sampling date and for each replicate, the aerial and root parts of the plants dedicated to these measures were cut, dried, and weighed. After measurement of the disease (symptoms and pathogen quantification as described below), the roots of plants grown for nucleic acid extraction were cut, washed in sterile water, sliced into small pieces, frozen in liquid nitrogen, lyophilized, and grounded with a FastPrep-24 (MP biomedicals). The dry root biomass was measured and the powder was kept at −80°C until nucleic acid extraction (DNA for pathogen quantification and RNA for RNA-seq analyses).

Disease was assessed at each sampling date after inoculation with *P. brassicae*. First, clubroot symptoms were evaluated by a disease index calculated with the scale previously described (Manzanares-Dauleux et al., 2000). Secondly, 1 μl of DNA extracted from root samples (see next paragraph) was used for quantitative PCR on the 18S gene to quantify the *P. brassicae* amount as previously described (Daval et al., 2020).

For statistical analyses between modalities of aerial and root biomasses, disease data, and 18S gene levels, linear models were used (LMM function “lmer,” package “lme4”) (Bates et al., 2015). Estimated Marginal Means (EMMeans) were calculated using the “emmeans” function of the “emmeans” package (Searle et al., 1980). Pairwise comparisons of EMMeans were performed (α = 5%), using the “cld” function of the “emmeans” package.

### Nucleic Acids Isolation From Roots

The lyophilized and ground roots from pooled plants of each genotype and each treatment (24, 12, and 6 plants at 14, 27, and 42 dpi, respectively) were used for nucleic acid extraction.

DNA was extracted and used for *P. brassicae* quantification by qPCR (Supplementary Table 1B) as previously described (Daval et al., 2020).

Total RNA was extracted from 20 mg of lyophilized powder with the Trizol protocol (Invitrogen). RNA purity and quality were assessed with a Bioanalyzer 2100 (Agilent) and quantified with a Nanodrop (Agilent).

### Library Construction and Illumina Sequencing

RNA-seq analysis was performed on RNA extracted from root tissues of both genotypes Y and HD, infected with resting spores of *P. brassicae* (eH isolate), and grown in the two different N conditions (N1 and N8) for four replicates, at 14, 27, and 42 dpi.

The preparation of Shotgun Random mRNA was conducted by Genoscreen (Lille, France). Library sequencing was conducted from both ends on an Illumina HiSeq4000 (Genoscreen, Lille, France) using 2 × 150 bp. The purified mRNA was fragmented and converted into double-stranded cDNA with random priming. Following end-repair, indexed adapters were ligated. The cDNA fragments of ∼350 bp were purified with AMPure XP beads and amplified by PCR to obtain the libraries for sequencing. The libraries were multiplexed (six libraries per lane) and sequenced, resulting in an expected average of 80 paired-end millions of reads per library (and then per sample). Raw reads of the 48 samples are available at the European Nucleotide Archive database system under the project accession number PRJEB44381 (samples ERS6263817–ERS6263820, ERS6263825–ERS6263828, ERS6263833–ERS6263836, ERS6263841–ERS6263844, ERS6263849–ERS6263852, ERS6263857–ERS6263860, ERS6263865–ERS6263868, ERS6263873–ERS6263876, and ERS6263881–ERS6263896).

### Read Mapping to the Reference Genome and Transcript Counting

The read quality was undertaken for the quality scores of Q28 and for the read length of 50 nucleotides using PrinSeq. The high-quality reads were aligned to the reference genome of eH *P. brassicae* (Daval et al., 2019) files using STAR 2.5.2a_modified. Non-default parameters were minimum intron length 10, maximum intron length 25,000, and maximum gap between two mates 300. Thanks to genome annotation files, the mapped sequencing reads were assigned to genomic features using featureCounts v1.5.0-p1 and counted. After filtering of the read counts below the threshold value (at least 0.5 counts per million in four samples), the count reads were then normalized with the TMM method. As the numbers of reads in the libraries were very different between each sampling time (due to the different infection rates and progression of the pathogen during the kinetic), the normalization was performed separately for each time. So, analyses of *P. brassicae* transcriptomes were specific to each sampling time, preventing the data comparison between the time points.

### Identification of Differentially Expressed Genes (DEGs) and Gene Co-expression Clustering Analysis

Analyses were performed using the R package AskoR developed in the laboratory^1^, allowing the integration of several packages widely used in gene expression in an automatic way. In this package, Edge R package was used for differential expression analysis and a false discovery rate (FDR) cutoff of 0.05 was applied to account for multiple testing correction. The DEGs with FDR ≤ 0.05 from specific comparison lists were selected for analysis. AskoR generated heat maps and Venn diagrams using the heatmap3 and the VennDiagram package, respectively. The clustering of gene expression profiles was performed, through AskoR, using CoSeq package (Rau and Maugis-Rabusseau, 2018; Godichon-Baggioni et al., 2019) by making groups of co-expressed genes on the EdgeR normalized counts. The UpSetR package was also integrated into AskoR for the visualization of intersecting sets of DEGs (Conway et al., 2017).

### Gene Ontology Term (GO-term) Enrichment

Gene Ontology enrichment analysis of DEGs was achieved with AskoR using TopGO R package (Alexa and Rahnenfuhrer, 2007) with weight01 algorithm, Fisher’s exact statistic test, and a nodeSize parameter set to 5 (to remove enriched GO-term with less than five genes in the genome). For each GO category (Molecular Function, Cellular Component, and Biological Process), the top 15 enriched GO-terms (*p*-value < 0.05), enrichment ratios (>2), and number of genes under each enriched GO-term were represented using the ggplot2 R package.

### Quantitative Real-Time PCR (RT-qPCR) Validation

The expression levels of 16 DEGs were determined by RT-qPCR to confirm the results of RNA-seq analysis. Total RNA from roots infected by *P. brassicae* was reverse transcribed with a set of two external RNA quality controls (Sabater-Munoz et al., 2006) as previously described (Daval et al., 2011, 2013). Seven hundred and fifty ng of total RNA were mixed with known quantities of the two external controls. Reverse transcription was carried out in 30 μl containing 375 ng of random primers, 1 × ImPromII reaction buffer, 3 mM MgCl_2_, 125 μM of each dNTP, 30 U of RNasin Ribonuclease Inhibitor, and 1.5 μl of ImProm-IITM (Promega). The following parameters were applied: 5 min at 25°C, 1 h at 42°C, and 15 min at 70°C. Reactions without RNA or without reverse transcriptase were performed as negative controls. The oligonucleotides designed with the Primer 3 software are described in Supplementary Table 1B. Quantitative PCR reactions (20 μl) containing 1 μl of cDNA, 0.4 μM of each primer, and 1 × SybrGreen I Master (Roche) were performed on the LightCycler1 480 Real-Time PCR System (Roche). The quantitative PCR profile consisted of an initial denaturation at 95°C for 5 min, followed by 45 cycles of 95°C for 15 s, and hybridization–elongation temperature for 40 s (Supplementary Table 1B). A dissociation stage was applied at the end of the PCR to assess that each amplicon generated was specific. Moreover, each specific amplicon was sequenced (Genoscreen, Lille, France) to confirm it corresponded to the expected sequence. The expression levels of transcripts were normalized using external RNA controls from four independent biological replicates, each with three technical PCR replicates. For this normalization, the expression levels of each quantified transcript were calculated from the *C*_*T*_ values (Daval et al., 2011). Using the geNorm software, an accurate gene expression normalization factor of qPCR data was calculated based on the geometric average of the two external controls *C*_*T*_. For each candidate gene and each sample, the ratio between the relative expression value and this normalization factor gave the corrected expression level of the gene. Data were analyzed using the ANOVA procedure of the R statistical analysis software. The correlation between the two sets of data corresponding to gene expressions obtained by RNA-seq and by RT-qPCR was determined using the Spearman rank correlation test.

## Results

### Modulation of Plant Clubroot Symptoms According to the Nitrogen Supply and the Host-Plant Genotype

**FIGURE 1:**
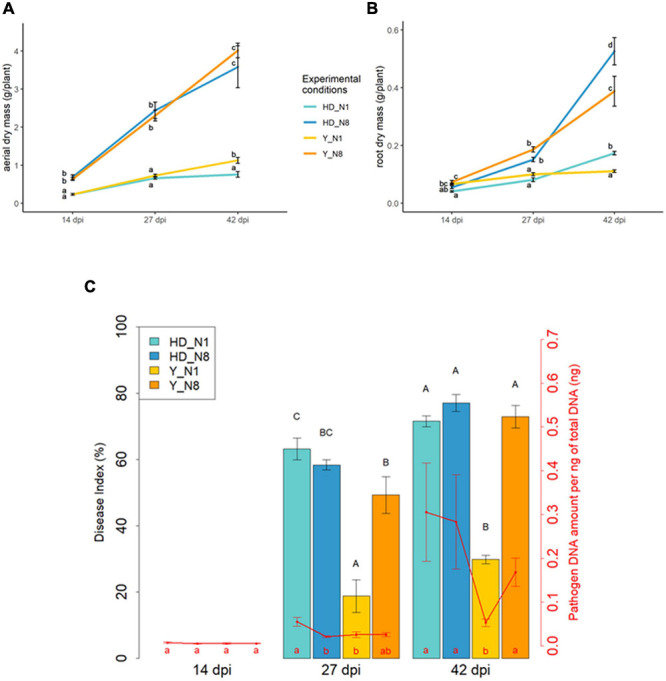
Influence of nitrogen supply on plant biomass and clubroot development. The dry aerial parts **(A)** and roots **(B)** were weighed for both inoculated genotypes (Y, Yudal and HD, HD-018) at different days post-inoculation (14, 27, or 42 dpi), and for each N condition supply (N1, 1 mM and N8, 8 mM nitrogen). The clubroot symptoms were estimated according to the disease index and the quantification of *P. brassicae* DNA by qPCR expressed as a ratio of the 18S DNA quantity relative to the total DNA **(C)**. Data are means of four biological replicates (6–24 pooled plants per time, per plant genotype, and per replicate), and error bars represent standard errors of the means. Means with different letters are statistically significantly different according to the analysis of variance test (*P* < 0.05).

The dry aerial parts of inoculated plants (Figure 1A) increased all over the entire experiment, especially in the N8 condition. For each N condition and each time-course point, no significant differences were measured between Y and HD genotypes. From 14 dpi, the plants grown in N8 displayed significantly higher aerial biomasses than those in the N1 condition, to reach in both genotypes an aerial dry weight 6–7 times greater in N8 than in N1 at the end of the experiment. Non-inoculated plants displayed comparable growth profiles to those of inoculated plants (data not shown) in function of time and N supply. The roots (Figure 1B) exhibited similar profiles to the aerial parts, except at 42 dpi in both N conditions where HD had a slightly higher weight than Y. Thus, there was no important effect of genotype but a high effect of N condition on the growth profile.

At each sampling time, disease severity was scored by determining the disease index (DI) and the DNA pathogen content (Figure 1C). The non-inoculated plants showed no symptoms and did not contain any clubroot pathogen according to our detection threshold (data not shown). In inoculated plants, the DI value (not yet measurable at 14 dpi) increased along the time points, but this increase was smaller for Y at N1 compared to the three other conditions. The DI was twice smaller (*p* < 0.05) in Y in N1 compared to HD in both N fertilization conditions and to Y in N8. The DNA pathogen content, well detectable from 27 dpi, followed almost the same pattern and increased over the time-course experiment. Although the reduction of symptoms was more pronounced between Y N1 and the three other conditions from 27 dpi, the DNA content was significantly lower only in Y N1 compared to HD N1 at 27 dpi, but significantly lower in Y N1 than all the three other conditions at 42 dpi.

### Overview, Mapping, and Validation of RNA-seq Data

The impact of N on *P. brassicae* transcriptome was investigated by RNA-seq analysis of inoculated roots. We obtained about 80 million reads by sample. The percentage of reads mapped to the eH reference genome increased with the experimental time course and was concordant with the observed increase in the pathogen amount during the infection (Supplementary Figure 1A). The standardized data per million counts were plotted on a PCA to estimate the variability of the biological experiments (Supplementary Figure 1B). Sixty-four percent of the variance were represented on the plot. The *x*-axis (55.48%) separated clearly the sampling time. The four replicates of each experimental condition were overall clustered together, which made it possible to validate our RNA-seq experiment. Pathogen gene expression’s profiles, described by heatmaps at each sampling time (Supplementary Figure 1C), were clustered at 14 dpi first by the N condition and then by the host genotype, and at 27 and 42 dpi first by the host genotype and then by the N supply condition.

A validation of the RNA-seq data was performed by RT-qPCR assay whose amplification conditions are described in Supplementary Table 1B. For this, 16 genes, which were significantly differentially expressed at least in one of the six main comparisons (described in Table 1) and which provided a large range of expression levels, were used to compare log2FoldChange values between RT-qPCR and RNA-seq experiments (Supplementary Figure 2). We found a moderate correlation (*r*^2^ = 0.48) between log2FoldChanges values of both types of experiments but a significant relationship according to Spearman correlation test (*p*-value = 6.11 10^–14^), confirming the validity of the RNA-seq data and a reliable expression result generated by RNA-seq.

**TABLE 1:** Number of *Plasmodiophora brassicae* DEGs for each comparison of interest.

**Infection stage**	**Comparison between conditions**					
	**Y N1 compared to Y N8**	**HD N1 compared to HD N8**	**Y N1 compared to HD N1**	**Y N8 compared to HD N8**	**Y N1 compared to HD N8**	**Y N8 compared to HD N1**
14 dpi	37	19	55	1	194	128
27 dpi	99	600	742	450	1985	316
42 dpi	318	5	119	140	613	544

*DEGs, differentially expressed genes; dpi, days post-inoculation; Y, Yudal genotype; HD, HD-018 genotype; N1, 1 mM nitrogen supply; N8, 8 mM nitrogen supply.*

### Global Overview of the Differential Gene Expression Profiles

Table 1 shows the number of DEGs in *P. brassicae* in the main comparisons between conditions of interest, combining host genotype and N supply. For both host genotypes, N input had a low impact on the *P. brassicae* transcriptome at 14 dpi with only 19 and 37 DEGs when HD and Y were host, respectively. Interestingly, for the later time-course points, *P. brassicae* displayed a different profile of DEGs between N conditions, depending on the host genotype with a high number of DEGs at 27 dpi when infecting HD (600 DEGs between N1 and N8) and a high number of DEGs at 42 dpi when infecting Y (318 DEGs between N1 and N8). When both factors (N and host genotypes) were considered, a gradual increase over time of the DEG number between Y N8 and HD N1 occurred with 128, 316, and 544 at 14, 27 and 42 dpi, respectively. Concerning the comparison Y N1 versus HD N8, it is interesting to note that the number of DEGs was high, especially at 27 dpi, with 1,985 DEGs corresponding to about 20% of the total genes expressed.

**FIGURE 2:**
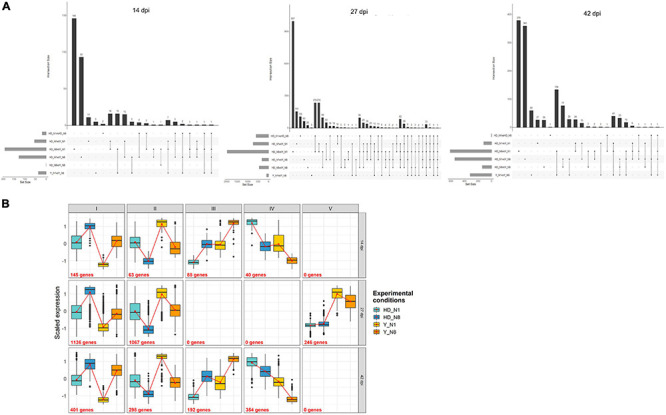
Global view of *Plasmodiophora brassicae* transcriptome profiles at the three time-course points. **(A)** Plot of intersections between sets of DEGs. The intersected lists of DEGs and their sizes (number of DEGs) are presented in the horizontal bars on the left. The connected lines among the lists represent their intersections. The vertical bars and associated numbers correspond to specific overlap of DEGs sets. The significance of gene expression changes was inferred based on an adjusted *p*-value < 0.05. **(B)** Co-expression clusters. CoSeq package was used to cluster expression data. The number of genes assigned to a particular cluster is indicated with the cluster name. *Y*-axis represents, for each gene, the number of centered reduced counts (previously filtered and normalized with EdgeR). DEGs, differentially expressed genes; Y, Yudal; HD, HD-018; N1, 1 mM N supply; N8, 8 mM N supply.

To further decipher the transcriptomic fingerprints of *P. brassicae* as a function of plant genotype and nitrogen supply, we plotted the intersections between the lists of DEGs for each of the six main comparisons of interest on an Upset graph (Figure 2A). Among the lists of DEGs whose total numbers are given in Table 1, the higher number of specific genes differentially expressed (not encountered in other comparisons) was found in the HD N8/Y N1 comparison, with 146, 997, and 379 specific DEGs at 14, 27, and 42 dpi, respectively. This was followed by the HD N1/Y N8 comparison that showed the largest number of specific DEGs, with 93 and 360 genes at 14 and 42 dpi, respectively. The proportion of DEGs in at least two comparisons relative to the total number of DEGs was 24% at 14 dpi (79 genes out of 336), 45% at 27 dpi (1,101 genes out of 2,449), and 31% (392 genes out of 1,245) at 42 dpi. Thus, on the other hand, at each time-course point, the DEGs were mostly specific for a given contrast.

A clustering of DEGs considering the genes differentially expressed in at least one comparison allowed description of the different expression profiles at each time point (Figure 2B). Profiles I and II were found at the three time-course points of the experimentation. They displayed similar expression in HD N1 and Y N8, but they differed in the expression of HD N8 compared to that of Y N1: this expression was higher in HD N8 than Y N1 in profile I but lower in profile II. Profiles III and IV were found at 14 and 42 dpi but not at 27 dpi. Profile III was characterized by the weakest expression in HD N1 and the strongest in Y N8, and profile IV was characterized by the opposite, and both profiles displayed intermediate and equal expression in HD N8 and Y N1. Gene expression in HD N8 and Y N1 therefore seemed to be decisive in determining the DEGs clustering. Profile V was specific of the time 27 dpi, with the lowest expression for HD N1 and HD N8, and the highest for Y N1 and Y N8. For this descriptive global overview of expression profiles, the lists of DEGs were not specifically extracted, because in total, they represented the complete list of DEGs of the experiment.

### Effect of the Nitrogen Supply Condition on the *P. brassicae* Transcriptome Independently of the Infected Host-Plant Genotype

The description of the DEG numbers between nitrogen supplies is depicted in Table 2. The number of DEGs between N1 and N8 for both genotypes was low at 14 dpi (52 genes) with almost 3/4 being underexpressed in N1 compared to N8. The largest number of DEGs was found at 27 dpi (645 genes in total), with approximately equal number of genes overexpressed at N1 (361 genes) and at N8 (284 genes). At the final time-course point, of the 321 DEGS in total, the majority were underexpressed at N1 (224 genes).

**TABLE 2:** Number of *Plasmodiophora brassicae* DEGs depending on the nitrogen supply.

	**Total**	**Underexpressed in N1 compared to N8**	**Overexpressed in N1 compared to N8**	**Common DEGs in both host genotypes**
14 dpi	52	37	15	4
27 dpi	645	361	284	54
42 dpi	321	224	97	2

*DEGs, differentially expressed genes; dpi, days post-inoculation; N1, 1 mM nitrogen supply; N8, 8 mM nitrogen supply.*

**FIGURE 3:**
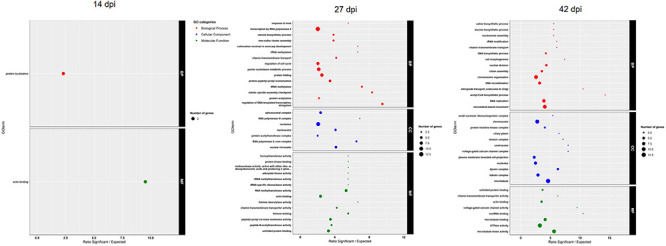
GO enrichment on DEGs lists according to the nitrogen effect at each time-course point. DEGs, differentially expressed genes; dpi, days post-inoculation.

A GO-term enrichment analysis was achieved from the 52, 645, and 321 DEGs at each sampling time (Figure 3) and the genes responsible for the enrichment of the main GO-terms, as well as their expression levels in each condition, are described in Supplementary Table 2. The GO-terms enriched at 14 dpi were involved in general cell divisions: protein localization, a crucial mechanism controlling access to and availability of all types of molecular interaction partners, and actin binding, contributing to many cellular structure, functions, and motility. In the same way, at 27 dpi, among the 645 DEGs linked to nitrogen supply, 19 genes were involved in mitosis-linked process (“actin binding,” “histone deacetylase activity,” “histone binding,” “isomerase activity,” and “acetyltransferase activity”) and 8 were involved in methyltransferase activity, a key component of genetic regulation. Interestingly, the molecular function “fucosyltransferase activity” was enriched at 27 dpi, and enzymes were classified on the basis of sequence similarities in the CAZy (carbohydrate-active enzymes) and were important for host–microbe interactions. Finally, at 42 dpi, many mechanisms involved in cell divisions and cell-signaling systems were enriched (“nucleosome assembly,” “DNA biosynthetic process,” “chromosome organization,” “DNA recombination,” “DNA replication,” or “microtubule-based movement”). The enriched molecular function “unfolded protein binding” was found, corresponding to a key cellular mechanism to restore protein homeostasis.

**FIGURE 4:**
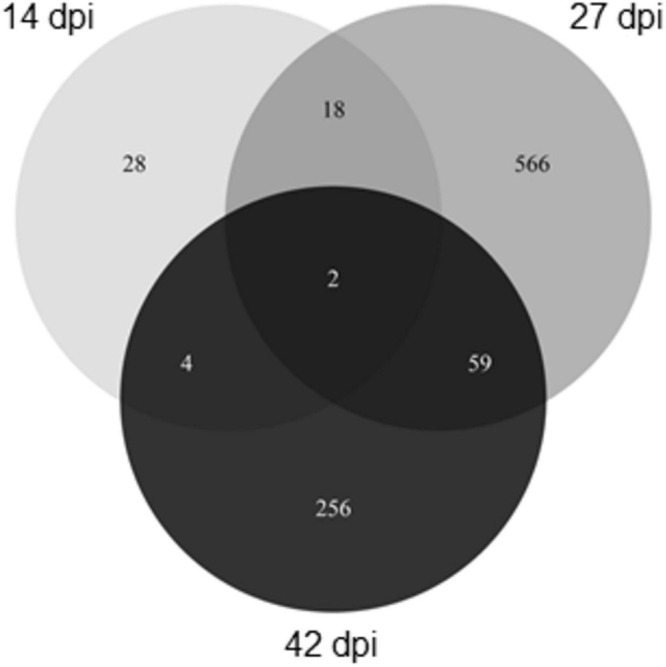
Number of *Plasmodiophora brassicae* DEGs between N supply conditions. The Venn diagrams show the number of significantly DEGs (*P* < 0.05) between N1 and N8 for both genotypes between sampling dates. DEGs, differentially expressed genes; dpi, days post-inoculation; N1, 1 mM nitrogen supply, N8, 8 mM nitrogen supply.

DEGs between N1 and N8 were globally specific for a given time (Figure 4): 28 genes were differentially expressed exclusively at 14 dpi, 566 genes (out of 645) exclusively at 27 dpi, and 256 genes (out of 321) exclusively at 42 dpi. At each time-course point, a small number of genes were differentially expressed between N1 and N8, concurrently in Y and HD: 4 genes at 14 dpi (i.e., 7.7%), 54 genes at 27 dpi (i.e., 8.4%), and 2 genes at 42 dpi (i.e., 0.6%) (Table 2). These 60 genes showed the same expression profile between N1 and N8 for both host genotypes.

To go further in the description of mechanisms potentially modulated by N supply, we have particularly studied the common sets of DEGs between N1 and N8 (among the complete list of DEGs between N1 and N8 described in Supplementary Table 3) whose expression profiles quantitatively varied in function of N conditions but independently of host genotype.

At 14 dpi (Supplementary Table 3A), among the four DEGs in both HD and Y, one corresponded to a hypothetical protein, one was overexpressed in N1 compared to N8 and encoded a “superoxide dismutase,” and two genes underexpressed in N1 compared to N8 encoded “pentatricopeptide repeat-containing,” whose putative function could be involved in growth.

At 27 dpi (Supplementary Table 3B), among the 54 common genes, 32 were related to hypothetical proteins. From the 22 remaining genes identified as potential known proteins, 18 were underexpressed in N1 compared to N8, as those encoded for “AGC kinase” involved in development, “ankyrin repeat” (two genes), “serine carboxypeptidase,” or “NUDIX hydrolase,” a domain present in “Nucleoside diphosphate-linked moiety X (NUDIX)” effectors, a group of secreted proteins identified in a broad range of pathogens, “4-pyridoxolactonase” (EC3.1.1.27) involved in the vitamin B6 degradation, several “heat shock proteins” and “disulfide isomerase” that can function as intra-cellular chaperones for other proteins and as part of cellular stress response, “ion transporter,” “cytochrome C oxidase,” or “methyltransferase” involved in many reactions such as cellular respiration, and “NEP1-interacting-like-2-isoform X1” (Pldbra_eH_r1s029g11071) known for their cytotoxicity in plants. The four genes overexpressed in N1 compared to N8 referred to “NHL-repeat binding protein” found in a large number of eukaryotic and prokaryotic proteins (like serine/threonine protein kinase in some pathogenic bacteria), a ubiquitous “transcription factor,” a “glutamine synthetase” that plays an essential role in the metabolism of nitrogen, and a “histone H2B” involved in mitosis.

Finally, at 42 dpi (Supplementary Table 3C), only two genes were commonly differentially expressed when both host genotypes were infected, one encoded a hypothetical and one underexpressed in N1 compared to N8 and encoded for “sporangia induced dynein heavy” playing a role in spore production.

### Nitrogen Supply Effect on the *P. brassicae* Transcriptome According to the Infected Host-Plant Genotype

**FIGURE 5:**
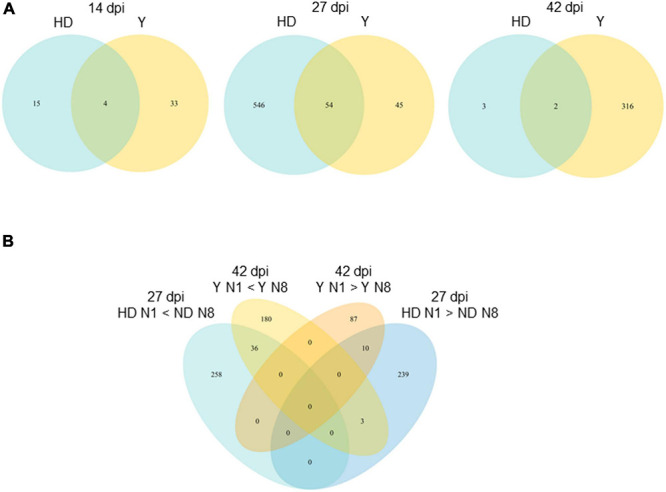
Number of *Plasmodiophora brassicae* DEGs between N1 and N8 according to the host genotype. The Venn diagrams show the number of significantly DEGs (*P* < 0.05) between N1 and N8 for each host *B. napus* genotype at each sampling date **(A)**, and for both genotypes between sampling dates **(B)**. DEGs, differentially expressed genes; dpi, days post-inoculation; Y, Yudal genotype; HD, HD-018 genotype; N1, 1 mM nitrogen supply; N8, 8 mM nitrogen supply.

The number of DEGs between N1 and N8 according to the host genotypes is presented in Figure 5A for each sampling date. At 14 dpi, Y has twice as many genes as HD, and even the total DEGs remained low. At 27 dpi, the majority of DEGs between N conditions were found when genotype HD was infected, with 600 genes out of 645 (93%), whereas in contrast at 42 dpi, the majority of DEGs were found when the infected genotype was Y, with 318 out of 321 (99%). The number of *P. brassicae* DEGs common to both infected genotypes was low, showing a nitrogen regulation that is dependent on the host-plant genotype. The functions of the genes identified at 27 or 42 dpi and described in the section above can thus finally be linked to the HD and Y genotypes, respectively.

A more in-depth analysis was performed on DEGs at 27 dpi when HD was infected and at 42 dpi when Y was infected, taking into account the over- or underexpression depending on the N condition (Figure 5B). In both categories of *P. brassicae* genes (those overexpressed and those underexpressed in N1 compared to N8), few genes were common between DEGs at 27 dpi in infected HD and at 42 dpi in infected Y. This showed that the difference in the number of DEGs was not due to a simple time lag in infection between the two genotypes, but to a difference in the response of *P. brassicae* to nitrogen supply as a function of host genotype and interaction time.

### Identification of *P. brassicae* Transcriptome Features Specific to Low-Nitrogen-Induced Resistance in Yudal

**FIGURE 6:**
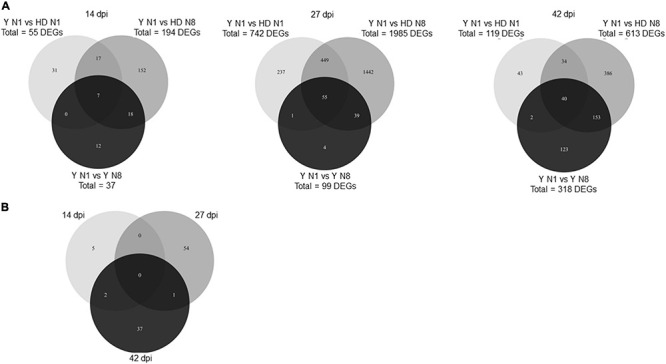
Number of *Plasmodiophora brassicae* DEGs between Y N1 and each of the other three conditions. **(A)** The Venn diagrams show the number of significantly DEGs (*P* < 0.05) between Y N1 and Y N8, or HD N1, HD N8 at each sampling date. **(B)** The Venn diagram focuses on the DEGs common between Y N1 and the other three conditions described at each of the three interaction time-course points (i.e., 7 genes at 14 dpi, 55 genes at 27 dpi, and 40 genes at 42 dpi). DEGs, differentially expressed genes; dpi, days post-inoculation; Y, Yudal genotype; HD, HD-018 genotype; N1, 1 mM nitrogen; N8, 8 nitrogen supply.

To better understand the potential underlying mechanisms related to the difference of the low infection level of Y in the N1 supply compared to the high symptom level in the other conditions (Y in N8 and HD in both N supplies), the specific DEGs between Y N1 and the other three conditions were studied at each interaction time (Figure 6A). The largest number of DEGs was found at 27 dpi with 2227 DEGs compared to 237 at 14 dpi and 781 at 42 dpi. For each time-course point, the greatest number of DEGs was found between Y N1 and HD N8, corresponding to both the N effect and the genotype effect, this contrast representing 78–89% of the total number of DEGs. Genes differentially expressed simultaneously between Y N1 and the three other conditions were only 7 at 14 dpi, 55 at 27 dpi, and 40 at 42 dpi, i.e., 102 in total, described in Supplementary Table 4. In addition, Figure 6B shows that these genes were mostly specific for each interaction time point since only 2 DEGs were common to 14 and 42 dpi, and 1 gene was common to 27 and 42 dpi. No gene common to all time-course points was differentially expressed between Y N1 and the three other conditions.

Among the 102 DEGs of Figure 6A, 52 were not identified (“hypothetical protein,” Supplementary Table 4), and 50 were identified by a GO-Term (Table 3 and Supplementary Table 4). Among these 50 genes, 9 were overexpressed and 41 were underexpressed in Y N1 condition.

**TABLE 3:** *Plasmodiophora brassicae* genes simultaneously differentially expressed in Y N1 compared to the three other conditions.

**Interaction time**	***P. brassicae* gene**	***P. brassicae* gene expression level**				**Description**
		**in Y_N1**	**in Y_N8**	**in HD_N1**	**in HD_N8**	
14 dpi	Pldbra_eH_r1s002g00869 Pldbra_eH_r1s008g04491 Pldbra_eH_r1s017g08107 Pldbra_eH_r1s026g10379	0.22 106.00 273.64 32.25	12.38 185.05 430.81 83.65	9.44 182.15 400.69 84.29	16.12 268.57 434.53 84.58	ATP GTP-binding protein kinase Kinase domain Chromodomain-helicase-DNA-binding 7 isoform X1 Vacuolar sorting-associated 13

27 dpi	*Pldbra_eH_r1s002g00900* Pldbra_eH_r1s003g02060 Pldbra_eH_r1s004g02133 Pldbra_eH_r1s005g02782 Pldbra_eH_r1s005g02887 Pldbra_eH_r1s006g03454 Pldbra_eH_r1s006g03599 Pldbra_eH_r1s006g03625 *Pldbra_eH_r1s008g04647* Pldbra_eH_r1s011g06084 *Pldbra_eH_r1s012g06550* Pldbra_eH_r1s014g07249 Pldbra_eH_r1s015g07600 Pldbra_eH_r1s017g08043 Pldbra_eH_r1s018g08464 Pldbra_eH_r1s018g08619 *Pldbra_eH_r1s019g08834* Pldbra_eH_r1s022g09660 Pldbra_eH_r1s027g10649 *Pldbra_eH_r1s028g10823* Pldbra_eH_r1s029g11027 Pldbra_eH_r1s029g11071 *Pldbra_eH_r1s033g11505* Pldbra_eH_r1s037g11877	2.17 1824.32 6.28 76.76 58.66 22.13 2839.60 79.35 2.33 81.67 91.93 118.64 12.42 620.67 312.47 7387.43 156.34 178.76 691.14 40.83 4.42 436.48 4.57 998.20	0 2679.49 12.74 124.45 107.09 35.08 4625.62 108.00 0.12 109.84 64.61 163.23 27.25 914.36 428.92 10149.25 108.15 293.35 1348.12 23.77 10.58 574.60 0.59 1806.81	0.36 2782.38 13.67 110.88 37.02 42.93 3867.11 102.69 0.08 116.44 63.40 204.29 23.66 841.54 446.17 10588.46 97.69 250.30 1151.69 23.22 16.86 592.03 0.97 2029.42	0 4241.63 29.82 175.18 104.68 52.57 7217.39 117.83 0 130.85 56.29 268.61 34.14 1154.60 589.06 14572.31 57.93 373.98 2022.38 18.69 17.56 894.01 0.66 3731.75	Regulator of chromosome condensation (RCC1) family AGC kinase Ankyrin repeat Adenosine triphosphatase Heat shock Ssa2 Ankyrin repeat Luminal-binding 5 30S ribosomal S16 Centrosomal POC5 (macronuclear) *S*-adenosylmethionine decarboxylase proenzyme-like General transcription factor IIH Iq domain-containing g Intraflagellar transport 122 homolog Serine carboxypeptidase family Ankyrin repeat-containing domain *S*-adenosylmethionine-dependent methyltransferase At5g38100 Glutamine synthetase FKBP-type peptidyl-prolyl cis-trans isomerase Disulfide isomerase Y-family DNA polymerase Tyrosine-kinase Src42A NEP1-interacting-like-2-isoform X1 Glycosyltransferase NUDIX hydrolase

42 dpi	Pldbra_eH_r1s001g00113 Pldbra_eH_r1s001g00127 Pldbra_eH_r1s001g00331 Pldbra_eH_r1s001g00618 Pldbra_eH_r1s001g00659 *Pldbra_eH_r1s002g00895* Pldbra_eH_r1s002g01266 Pldbra_eH_r1s002g01288 Pldbra_eH_r1s004g02319 Pldbra_eH_r1s005g02870 *Pldbra_eH_r1s008g04609* Pldbra_eH_r1s009g05132 Pldbra_eH_r1s009g05217 Pldbra_eH_r1s011g06038 Pldbra_eH_r1s012g06458 Pldbra_eH_r1s013g06611 Pldbra_eH_r1s014g07146 Pldbra_eH_r1s015g07419 Pldbra_eH_r1s015g07500 Pldbra_eH_r1s015g07561 Pldbra_eH_r1s021g09305 *Pldbra_eH_r1s053g12569*	73.64 129.25 243.77 187.01 230.32 377.54 140.81 189.29 358.78 28.51 26.03 0.09 59.34 70.99 162.17 257.85 43.88 42.39 111.71 197.19 369.19 126.38	102.51 171.67 412.32 279.84 325.73 272.52 228.54 351.64 517.01 73.95 16.48 1.21 111.47 93.60 215.60 405.35 96.57 93.37 216.12 323.11 639.38 96.04	97.98 162.73 411.74 263.76 331.44 259.27 215.10 308.38 526.94 63.75 16.08 1.07 136.50 101.08 238.30 359.86 73.44 91.10 204.77 293.71 549.37 90.53	97.23 167.09 407.37 258.39 360.67 251.73 231.26 326.88 477.75 67.87 13.09 1.28 121.52 93.24 212.49 391.28 88.94 96.21 202.75 293.49 617.65 87.83	Centrosomal of 120 kDa-like Like subfamily c-member-21 Centrosomal of 290kDa-isoform X5 DNA topoisomerase 1 beta Chromosome segregation L-isoaspartate (D-aspartate) *O*-methyltransferase-like PIN2 TERF1-interacting telomerase inhibitor-1 Suppressor SRP40 Calreticulin precursor Viral A-type inclusion PAS domain S-box CMGC CDKL kinase Histone H1 Katanin p60 ATPase-containing subunit A-like Splicing factor 3a subunit 3 Nucleolar NOP5 Rad52 22 double-strand break repair Motor domain-containing Pre-rRNA-processing esf1 Eukaryotic rRNA processing Nucleolar 56-like Protein BUD31 homolog

*Genes underexpressed or overexpressed in Y N1 compared to the three other conditions are in normal font or in italics, respectively.*

At 14 dpi, the four identified genes were underexpressed in Y N1 and encoded an “ATP GTP-binding protein kinase” and a “kinase domain” involved in cellular signalization, a “chromodomain-helicase-DNA-binding 7 isoform X1” playing a role in DNA replication, and a “vacuolar sorting-associated 13,” a protein of unknown function.

At 27 dpi, many DEGs between Y N1 and the three other conditions (most of them underexpressed in Y N1) were involved in general metabolic pathways, such as: (i) development, cell growth and proliferation (“regulator of chromosome condensation,” “AGC kinase,” “centrosomal POC5,” “30S ribosomal S16,” “*S*-adenosylmethionine decarboxylase proenzyme,” “intraflagellar transport 122 homolog,” and “Y-family DNA polymerase”), (ii) energy metabolism or cellular respiration (“adenosine triphosphatase,” “transcription factor IIH,” and “*S*-adenosylmethionine-dependent methyltransferase”), (iii) chaperones as part of cellular stress response (“heat shock Ssa2,” “disulfide isomerase” “luminal-binding 5,” and “FKBP-type peptidyl-prolyl *cis-trans* isomerase”), and (iv) signaling pathway (“iq domain-containing g,” and “tyrosine-kinase Src42A”). Such gene expression profiles related to pathogen development were concordant with the lower clubroot symptoms observed in Y under N1 supply. Interestingly, genes potentially involved in the pathogenicity of *P. brassicae* (such as putative effectors) or causing cytotoxicity in the plant were differentially underexpressed in Y N1 (“ankyrin repeat,” “serine carboxypeptidase,” “NUDIX hydrolase,” and “NEP1-interacting-like-2-isoform X1”). In contrast, one gene encoding a potential pathogenicity facilitator (“glycosyltransferase”) was overexpressed in Y N1. Finally, it is essential to underline that a gene encoding a “glutamine synthetase,” a key enzyme for nitrogen metabolism involved in ammonium assimilation into amino acids, was overexpressed in *P. brassicae* in Y N1 condition compared to the three other conditions.

At 42 dpi, in the same way as at 27 dpi, many genes underexpressed in Y N1 were linked to mechanisms of growth and development of the pathogen (“centrosomal of 120 kDa-like,” “centrosomal of 290 kDa-isoform X5,” “DNA topoisomerase 1 beta,” “chromosome segregation,” “PIN2 TERF1-interacting telomerase inhibitor-1,” “suppressor SRP40,” “histone H1,” “Katanin p60 ATPase-containing subunit A-like,” “splicing factor 3a subunit 3,” “nucleolar NOP5,” “Rad52 22 double-strand break repair,” “motor domain-containing,” “pre-rRNA-processing esf1,” “eukaryotic rRNA processing,” “nucleolar 56-like,” “protein BUD31 homolog,” “viral A-type inclusion,” and “like subfamily c-member-21”), to signaling pathway (“CMGC CDKL kinase”), or related to stress response (“calreticulin precursor”). On the other hand, one gene putatively associated to repair/degradation of damaged proteins [“L-isoaspartate(D-aspartate) *O*-methyltransferase-like”] and one gene connected to the stress response (“PAS domain S-box”) were overexpressed in Y N1 condition compared to the three other conditions displaying higher infection level (Y N8, HD N1, and HD N8).

## Discussion

Nitrogen fertilization plays an important role in the outcome of the plant–pathogen interaction and thus in the development of the plant diseases. To explain this, it is wise to hypothesize that nitrogen can play a role on the defense mechanisms of the plant and/or on the development of the pathogen. Low-nitrogen conditional resistance was previously reported in Y (Laperche et al., 2017; Aigu et al., 2018). The genetic analysis of this trait in a Darmor × Yudal progeny had highlighted a quantitative genetic control, and the existence of at least two low-nitrogen conditional resistance loci. To better understand those phenomena and the clubroot modulation by N supply, it was also important to get a better idea how plant nitrogen supply can affect the physiology of *P. brassicae*, and if this influence could be different according to the host plant genotype. Thus, in the present work, we used a transcriptomic approach to investigate the pathogen molecular mechanisms that can underline the observed different levels of infection severity according to the presence of this low-nitrogen conditional resistance related to host-plant genotype. In this study, we aimed at focusing on the pathogen transcriptome including in an original way simultaneously different factors to be studied: nitrogen, plant genotypes, and interaction kinetics.

For this, *B. napus* cultures were conducted under two fertilization regimes, in which the nitrogen was supplied as nitrate, preferentially taken up by oilseed rape (Arkoun et al., 2012), and not as ammonium. As potassium and calcium can have effects on clubroot disease development (Dixon, 2009), the concentrations of these elements have been adjusted between the two nutrient solutions, making sure that the comparisons between N1 and N8 only focused on nitrate content. Finally, as the experiments were performed in potting soils and not in natural soils, biological interactions between nitrogen supply and potential soil microorganisms were limited, allowing accurate and controlled monitoring of the nitrogen conditions.

First, we confirmed that the low-nitrogen conditional resistance in Y was absent in HD, consistent with the data obtained in Laperche et al. (2017) and Aigu et al. (2018). Furthermore, in the present work, we showed that the low-nitrogen conditional resistance in Y resulted not only in lower symptoms but also in lower pathogen content in root tissues. Both genotypes had similar root and aerial growth profiles for a given nitrogen condition, except a very slightly less root growth in Y compared to HD in both nitrogen conditions. Moderate differences in root growth were already described in other genotypes of *B. napus* that can display different root developmental patterns (Daval et al., 2020). Both Y and HD displayed significantly lower growth at N1 than at N8, showing that N1 was a nitrogen constraint condition, as expected. The control non-inoculated plants displayed similar profiles of aerial and root weights, with no differences between genotypes, and significantly lower growth at N1 than at N8 for both genotypes (data not shown). Decreases in symptoms and pathogen content specific to Y N1 (and not to HD N1) were therefore not simply due to the impact of low nitrogen fertilization on plant growth.

*P. brassicae* transcriptome was influenced by host-plant nitrogen supply, but this modulation was very much dependent on host-plant genotype. Although numerous other studies have reported the influence of abiotic factors on pathogen gene expression (Fagard et al., 2014; Zarattini et al., 2021), probably few of them have reported the need for cautious multi-genotype evaluation before drawing general conclusions. The regulation of only 102 of *P. brassicae* genes was clearly associated to the low-nitrogen conditional resistance (i.e., to low rate pathogen growth in infected roots).

The modulation of clubroot disease could be explained by the influence of plant N nutrition on many factors involved in the different steps of the pathogen epidemiological cycle. The time-course study of host–pathogen interactions thus allowed us to highlight how relationships between *P. brassicae* and N could be established throughout the pathogen life cycle. Depending on the pathogen development stage (and thus the considered time-course point), we will discuss mechanisms by which nitrogen supply could modulate infection. These mechanisms could include (i) the availability of N compounds for exploitation by the pathogen, the quantity and nature of host N-based substrates acquired by the pathogen, particularly in relation to the obligate biotrophic character of *P. brassicae*; (ii) the metabolic pathways known to be specifically expressed at the primary or the secondary phase of the cycle; and (iii) the pathways and molecules involved in the pathogenicity, virulence, or aggressiveness of the pathogen. We thereafter discuss how this specific set of genes suggests a reduced expression of putative effectors and reduced metabolic and cell growth activities.

At 14 dpi, the N effect on *P. brassicae* transcriptome was moderate, particularly regarding the DEGs between N1 and N8 that were common to both genotypes, or the DEGs between Y N1 and the three other conditions (only seven genes for this last comparison). The gene coding for a “superoxide dismutase” was overexpressed in N1 compared to N8 whatever the host-plant genotype. The role of this enzyme has not been rigorously investigated, but it has been speculated to be important in the pathogenesis of bacterial and fungal infections (Cox et al., 2003). It is as if at this early stage of interaction, *P. brassicae* would attempt to infect more actively *via* this pathway to counteract the N restriction. Other DEGs at 14 dpi (“ATP GTP-binding protein kinase,” “kinase domain,” “chromodomain-helicase-DNA-binding CHD,” and “pentatricopeptide repeat-containing”) were underexpressed in N1 and were mainly involved in basal cellular processes, including metabolism, transcription, DNA repair, cell cycle progression, cytoskeletal rearrangement, and differentiation. The CHD proteins have thus been identified in a vast array of organisms as diverse as protists, plants, amphibians, and mammals, and a common theme for the main role of CHD proteins appears to be linked to their chromatin-remodeling activity (Hall and Georgel, 2007). Pentatricopeptide repeat proteins, found in all eukaryotes including protists, are RNA binding proteins with functions in organelle RNA metabolism. In a protist, they were shown to be involved in growth (Manna et al., 2013). At this early stage of interaction corresponding to the primary phase of infection, the carbon metabolism was particularly active (Schwelm et al., 2015a, b; Bi et al., 2016) but seemed to be weakly modulated by the N supply conditions.

All in all, at 14 dpi, N restriction had weak effects on *P. brassicae*, apart from a decrease in the expression of some genes involved in the growth of the pathogen. This is consistent with the fact that at this early stage, the resources allocated by the plant to the pathogen were not yet limiting because the plants showed equivalent levels of development regardless of the N treatment: differences in N resources were not yet very differentiated between treatments at this stage. The two metabolisms, nitrogen and carbon, which are intertwined and co-regulated (Nunes-Nesi et al., 2010), were therefore still little impacted by nitrate input.

At the 27 dpi interaction time, interesting regulations of *P. brassicae* gene expression seemed to occur depending on the N supply and the host plant genotype.

First, genes linked to growth and development were particularly underexpressed in Y N1. For instance, the “AGC-kinase” gene was described as required for development, invasion, and survival of the protist *Plasmodium falciparum* (Arencibia et al., 2013). In the fungus *Flammulina velutipes*, the “*S*-adenosylmethionine-dependent-methyltransferases,” which can transfer a methyl group to many kinds of biological molecules, play crucial roles in many important biological processes, such as small-molecule biosynthesis, and are involved in the fungal stipe elongation (Huang et al., 2019).

Other underexpressed genes in Y N1 were linked to a pathogen with a lower capacity of exploiting available nutrients and N compounds from infected plant cells. This was, for example, the genes coding for “ion transporters” or for “cytochrome-*c*-oxidase” linked to cellular respiration, or for “4-pyridoxolactonase,” involved in the degradation of vitamin B6 whose levels respond to an interaction with nitrogen metabolism (Mooney et al., 2009; Colinas and Fitzpatrick, 2016). This decrease in the capacity of *P. brassicae* to use nitrogen-related resources would partly explain the lower growth of the pathogen in the N1 condition.

Furthermore, in an interesting way, at 27 dpi (corresponding to the secondary phase of infection), many *P. brassicae* genes potentially involved in pathogenicity were underexpressed in N1 compared to N8. The molecular function “fucosyltransferase activity” was enriched, with an underexpression at N1 in both genotypes compared to N8 for the “alpha-1;3-fucosyltransferase” gene. The fucose-containing oligosaccharides on the cell surface of some pathogenic bacteria are thought to be important for host–microbe interactions and to play a major role in the pathogenicity of bacterial pathogens (Kajiwara et al., 2012). In the human pathogen *Toxoplasma gondii*, an obligate intracellular protist, a fucosyltransferase was shown to affect protein expression and virulence (Bandini et al., 2020). Other pathogenicity-related genes were particularly underexpressed in Y N1 compared to the three other conditions. This was the case for genes encoding “ankyrin repeat,” which are protein domains frequent in all Rhizarian (Glöckner et al., 2014), and present in effector candidates of plant pathogens (Pan et al., 2008). Ankyrin domains have been identified as mediators of protein–protein interactions, a key process involved in the interplay between effector–receptor interactions (Voronin and Kiseleva, 2008). In *P. brassicae*, this domain was detected in potential effectors and enriched in predicted proteins of its secretome (Schwelm et al., 2015a, b). A gene encoding “carboxypeptidase” was also underexpressed in Y N1. Many pathogenic fungi secrete serine carboxypeptidases, and this family of enzyme could be involved in the pathogenicity process (Monod et al., 2002; Lopez-Llorca et al., 2010). In *P. brassicae*, such proteases were also enriched in secretome (Schwelm et al., 2015b), suggesting a role in infection. In addition, genes of the “NUDIX” family were underexpressed in N1 condition for both plant genotypes, but especially when Y was the host-plant genotype. These pathogenicity-related genes, found in many phytopathogenic organisms (Dong and Wang, 2016), were previously described as overexpressed in conditions where clubroot symptoms were high (Daval et al., 2020). This suggests that this gene family may play an important role in the infectious process of *P. brassicae* and disease progression and strengthens the hypothesis that it represents relevant pathogenicity family genes. Another gene underexpressed in N1 encoded a “disulfide isomerase,” known as a molecular chaperone and component of signal-transduction pathways that is involved in fungi in the secretory pathway (Ngiam et al., 2000) and plays an important virulence role during plant infection by pathogens (Meng et al., 2015). Moreover, the “NEP1-interacting-like-2-isoform X1” was also underexpressed in N1, and even more markedly in Y N1. The “Necrosis and Ethylene-inducing Protein” (NEP)-like proteins are secreted by a wide range of plant-associated microorganisms (bacteria, fungi, and oomycetes); they are known for their cytotoxicity in dicot plants that leads to the induction of rapid tissue necrosis and plant immune responses, and they act as a microbe-associated molecular pattern (MAMP) (Oome et al., 2014). In fungi and oomycetes, NEP-proteins are especially present in species interacting with plants, and predominantly in species that display a hemibiotrophic, necrotrophic, or obligate biotrophic lifestyle on plants (Qutob et al., 2006; Lenarčič et al., 2019). Functional studies showed that NEP-proteins contribute to the virulence and pathogenicity of pathogenic microorganisms (Bae et al., 2006; Oome and Van Den Ackerveken, 2014; Levin et al., 2019). Such functional studies that characterize the gene coding for NEP and its role in infection are therefore now needed in *P. brassicae*.

On the contrary, few genes were overexpressed in Y N1. This was the case, for example, of the gene coding for a “glycosyltransferase.” In a fungal pathogen of wheat, a gene encoding a predicted “glycosyltransferase” was found to be involved in virulence by its role in extending surface hyphae (King et al., 2017). A similar role in *P. brassicae* is not known. Finally, and interestingly at 27 dpi, a gene encoding a “glutamine synthetase” was specifically overexpressed in Y N1 compared to three other conditions. “Glutamine synthetase” is an enzyme that plays an essential role in the metabolism of N by catalyzing the condensation of glutamate and ammonia to form glutamine as the principal nitrogen source for protein and nucleic acid biosynthesis. The glutamine synthetase activity, involved in the detection of nitrogen starvation (Sauer et al., 1999), is described as negatively affected by high N supply and dependent on the type of nitrogen source (Ferreira et al., 2015). In our study, the nutrient solution provided nitrogen as NO_3_, while the substrate of “glutamine synthetase” is NH_3_. This means that the “nitrate reductase” and the “nitrite reductase,” whose upstream “glutamine synthetase” activities produce NH_3_ from NO_3_, must also be highly active in Y N1. By overexpressing in part this pathway under N stress conditions, *P. brassicae* could attempt to compensate for the nitrogen deficiency.

The time-course point 27 dpi thus showed that many genes were under-expressed in N1 compared to N8. Despite a significant reduction in symptoms in Y N1 compared to the other three conditions, the number of DEGs specifically differentially expressed between Y N1 and these other conditions was low (only 55), but with interesting putative functions. The main pathways affected by N starvation when Y was the plant genotype were involved in the growth and pathogenicity mechanisms of *P. brassicae*, which was consistent with the observed symptoms and levels of disease. Inhibition of *P. brassicae* development and pathogenicity appeared to occur at 27 dpi, although at this time, the differences in pathogen DNA amounts between Y N1 and the three other conditions were not yet marked (the symptoms were). Thus, this gene underexpression at 27 dpi in Y N1 would explain the marked differences in pathogen DNA amounts at 42 dpi. The mechanisms by which the Y genotype limited the infection by *P. brassicae* specifically in N1 will have to be elucidated by functional studies on the candidate genes found in this study such as “NUDIX,” “carboxypeptidase,” and “NEP-proteins.”

At 42 dpi, many genes involved in growth, development, signaling pathway, or stress response were still underexpressed in Y N1, concordant with the lower infection in this condition. For example, the gene encoding “sporangia induced dynein heavy” and underexpressed in N1 was shown as important in flagella formation and zoospore motility in the genus *Phytophthora* (Narayan et al., 2010). Among the genes overexpressed in N1, one encoded “L-isoaspartate (D-aspartate) *O*-methyltransferase,” described as crucial for maintaining cellular integrity and increasing cell survival under physiological stresses (Banerjee et al., 2015). Another gene involved in stress response was overexpressed in Y N1, the “PAS domain S-box,” which is a signaling domain widely distributed in proteins from members of prokaryotes and eukaryotes, and whose functions are to sense stimuli (Taylor and Zhulin, 1999). Then at 42 dpi, *P. brassicae* was in a phase during which it felt the stimulus of N starvation and had a weak growth in Y N1.

## Conclusion

The description of the molecular response of *P. brassicae* during its interaction with two *B. napus* genotypes under different nitrogen supplies shed light on candidate genes that behave as growth and pathogenicity-related, some of which were previously reported. The successful colonization and growth of the pathogen was related to its ability to draw resources from the plant, depending on the host genotype, in connection with its obligate biotrophic features. Moreover, the mechanisms of infection *via* potential secreted effectors were regulated by N supply, again depending on the host genotype. The results obtained herein need to be completed by the study of *B. napus* molecular mechanisms regulated by nitrogen, by studying more precisely the mechanisms underlying the plant genotype-specific response. An increased understanding of the molecular responses to nitrogen is critical to reduce input costs, to minimize the potential environmental impacts of nitrogen fertilizer that has been used to optimize yield, and so to further improve agricultural sustainability.

## Data Availability Statement

The datasets presented in this study can be found in online repositories. The names of the repository/repositories and accession number(s) can be found below: https://www.ebi.ac.uk/ena, PRJEB44381.

## Author Contributions

SD, KG, MM-D, YA, and AG were involved in the conceptualization of the project, study design, critical inputs, and finalization of the manuscript. CL and MH were involved in wet lab experiments. KG and SD were involved in bio-informatics analyses and data compilation, and wrote the manuscript. MM-D, YA, and AG advised on the manuscript. All the authors contributed to the article and approved the submitted version.

## Conflict of Interest

The authors declare that the research was conducted in the absence of any commercial or financial relationships that could be construed as a potential conflict of interest.

## Footnotes

1 https://github.com/askomics/askoR

## Supplementary Material

Supplementary Figure 1 Overview of the *Plasmodiophora brassicae* transcriptome samples.

Supplementary Figure 2 Validation of RNA-seq approach by comparison of RT-qPCR and RNA-seq data.

Supplementary Table 1 Nutrient solutions and primers used in this study.

Supplementary Table 2 Description of the gene subsets responsible for the driving GO-term enrichments between the N supply conditions for each time.

Supplementary Table 3 Description of the *Plasmodiophora brassicae* DEGs (Differentially Expressed Genes) between N supply conditions for both host plant genotypes.

Supplementary Table 4 Description of the *Plasmodiophora brassicae* DEGs (Differentially Expressed Genes) between Y N1 and each of the other three conditions.
