# Supplementary material for: Nitrogen Supply and Host-Plant Genotype Modulate the Transcriptomic Profile of Plasmodiophora brassicae
Source: Front Microbiol. 2021 Jul 8;12:701067. doi: 10.3389/fmicb.2021.701067 (PMC8298192; doi:10.3389/fmicb.2021.701067)
Supplement: Supplementary Figure 1 — Overview of the Plasmodiophora brassicae transcriptome samples. [file Data_Sheet_1.PDF]

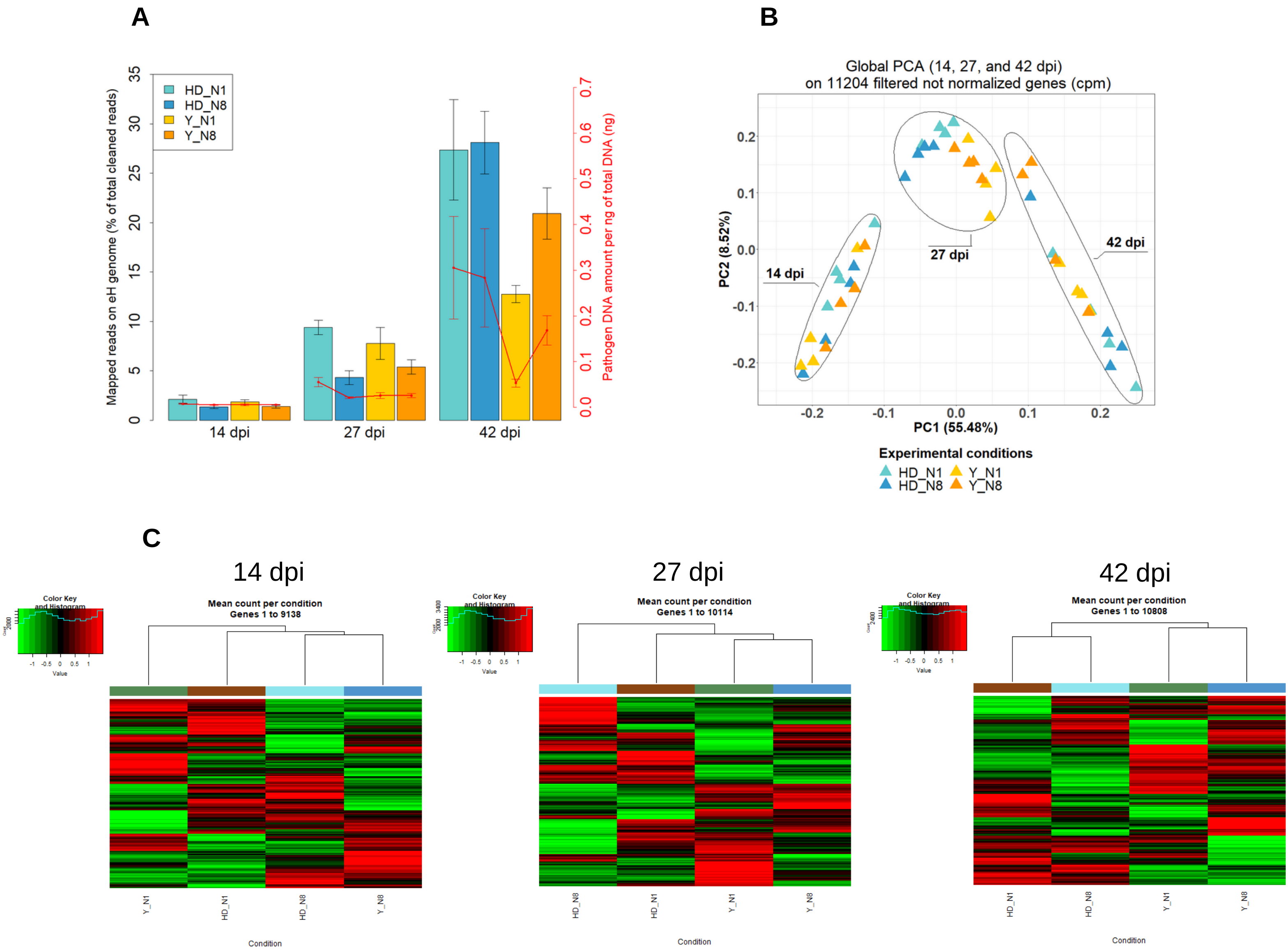

S1 Figure. Overview of the *Plasmodiophora brassicae* transcriptome samples.

A. Reads mapped to the eH reference genome.

B. Estimation of biological variations by Principal Component Analysis (PCA) of all the transcript profiles. X-axis represented the variance on the first axis of the PCA, and the second was picted on the y-axis.

C. Heatmaps of *P. brassicae* gene expression based on normalized data of expression values for each sampling time. The heatmaps are based on total reads counts for *P. brassicae* at 14, 27, or 42 dpi, for the N1 and N8 conditions, the two plant genotypes (Y and HD), and correspond to the mean of the four replicates.

Y, Yudal; HD, HD-018; N1, 1 mM nitrogen; N8 8 mM nitrogen; dpi, days post-inoculation.
