# Supplementary material for: Nitrogen Supply and Host-Plant Genotype Modulate the Transcriptomic Profile of Plasmodiophora brassicae
Source: Front Microbiol. 2021 Jul 8;12:701067. doi: 10.3389/fmicb.2021.701067 (PMC8298192; doi:10.3389/fmicb.2021.701067)
Supplement: Supplementary Figure 2 — Validation of RNA-seq approach by comparison of RT-qPCR and RNA-seq data. [file Data_Sheet_2.PDF]

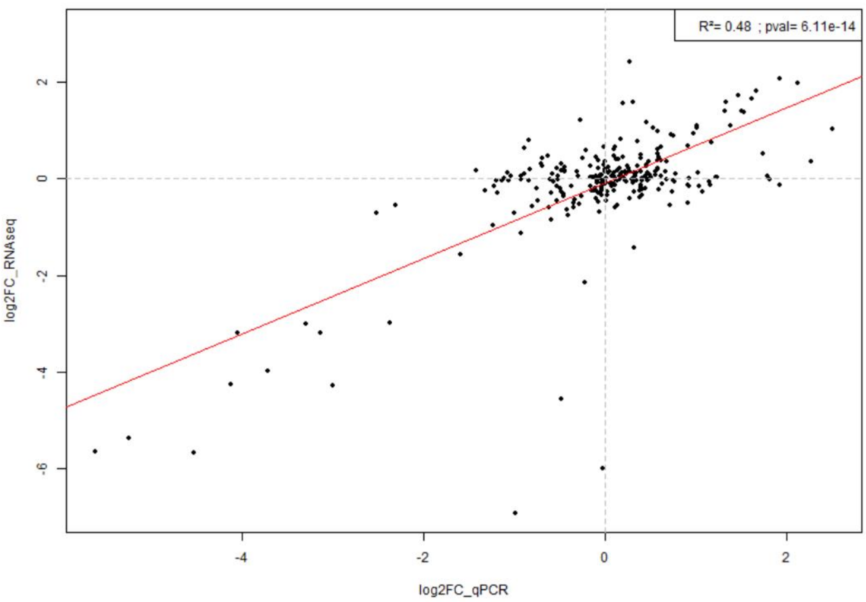

S2 Figure. Validation of RNA-seq approach by comparison of RT-qPCR and RNA-seq data.
The correlation was calculated between log2FoldChange of quantitative real-time PCR (x-axis) and RNAseq (y-axis) on 16 selected genes in the six main contrasts at the three kinetic times (a total of 258 of the 288 possible measurements).
