# Supplementary material for: Nitrogen Supply and Host-Plant Genotype Modulate the Transcriptomic Profile of Plasmodiophora brassicae
Source: Front Microbiol. 2021 Jul 8;12:701067. doi: 10.3389/fmicb.2021.701067 (PMC8298192; doi:10.3389/fmicb.2021.701067)
Supplement: Supplementary Table 1 — Nutrient solutions and primers used in this study. [file Table_1.DOCX]

S1 Table. Nutrient solutions and primers used in this study.

A. Composition of nutrient solutions.

|  | N1 solution | N8 solution | Both solutions were modified from the solution proposed by Hoagland, in order to adjust nitrogen concentration, either to 1 mM or to 8 mM. Bold characters indicate elements for which the nitrate concentration changed between the two nutrient solutions. |
| --- | --- | --- | --- |
| *Macro-elements (mM)*  KNO_3_  KH_2_PO_4_  MgSO_4_, 7H_2_O  CaCl_2_, 2H_2_O  Ca(NO_3_)_2_, 4H_2_O  KCl | **1**  0.5  1  2.5  **-**  2 | **3**  0.5  1  -  **2.5**  - |  |
| *Micro-elements (µM)*  MnSO_4_, H_2_O  ZnSO_4_, 7H_2_O  CuSO_4_  H_3_Bo_3_  Na_2_MoO_4_, 2H_2_O  FeEDTA | 10  1  0.5  30  1  27 | |  |

B. Primers and amplification conditions.

| Targeted gene | Forward primer 5’-3’ | Reverse primer 5’-3’ | Expected amplicon size (bp) | Annealing temperature (°C) |
| --- | --- | --- | --- | --- |
| Wn1 (Sabater-Muñoz et al., 2006)  Wn2 (Sabater-Muñoz et al., 2006)  18S  Pldbra_eH_r1s001g00344  Pldbra_eH_r1s001g00365  Pldbra_eH_r1s003g01648  Pldbra_eH_r1s005g02917  Pldbra_eH_r1s007g04056  Pldbra_eH_r1s008g04757  Pldbra_eH_r1s012g06299  Pldbra_eH_r1s014g07110  Pldbra_eH_r1s014g07238  Pldbra_eH_r1s016g07699  Pldbra_eH_r1s016g07790  Pldbra_eH_r1s017g08202  Pldbra_eH_r1s018g08619  Pldbra_eH_r1s023g09728  Pldbra_eH_r1s024g10144  Pldbra_eH_r1s061g12660 | TCATTTCCTTCTGGGCATTC  GTAACGTCGTCCCACTTTCAAT  TTGGGTAATTTGCGCGCCTG  CGACACCCTTACGTTTCGCA  AACCTGTTCGACACGTTCCG  ATGATTCCGATTAACCCGC  AAGGCGAATGTGATGGCGAT  GTCCTCGTCTGTGTTTCGCG  GCAACAGCGATTCGATCTGC  GACCCTGAGCTCATCGAACG  CGGCATCATCGACGATCAGG  GATGACGGCAAGGTGTACG  TGGTGGAACTTGACATCAGC  GCCGTTCTTCTCCATGACCA  TCACCCACCACATGATCCAC  GACTCGCCAGATGTTTTCCA  GCGACTGTTCCTGTCCGAAG  CCATTGTCGTCCCCTTCTGC  ACGTGAACATTCCAGCTCGC | GCACAAAACACTGCCATCAA  CTAACGTCGGGCGTAAACTAAT  CAGCGGCAGGTCATTCAACA  TCGATCTCCTGGACGATGCC  AACCTGTTCGACACGTTCCG  CCTGCCAGTTGTTACCTTGG  CTGGTTTGGGTGCAGGGC GATGTTGTGGGCGTTCAGGT  AAGAACGACGCTTTCATGCC  CAAGGCCCTTCTGCTTCTCG  GGCAACCTGTTCATCGCTGA  CTTCCAGGGCGAACATATCC  GACCGCGATATATTCCCGTC  GAAAGCAGAACAACAGCCGG  TGCCAAACATGTAGCCGAGT  GATCCGGATATGGCGATGTC  AGTCACGCTGCTGTACTTGC  CACCGTTCCAACTCTCGACC  ATTGCGCCCATTACTCACGG | 146  140  164  171  151  223  162  188  154  154  214  244  173  228  161  186  184  153  175 | 61  61  64  58  66  64  68  68  68  66  64  68  68  66  66  66  60  66  66 |

|  |  |
| --- | --- |
|  |  |

Wn1 and Wn2 are external RNA quality controls for RT-qPCR.
